# Endoscopic transcanal coblation excision of glomus tympanicum: a novel technique

**DOI:** 10.1007/s00405-024-08660-7

**Published:** 2024-04-30

**Authors:** Mohammed Abdelbadie Salem, Mahitab Ghoneim, Sally Sameh Ahmed, Ahmed Elsobki, Ahmed Abdoo Elzhzahy, Ahmed Hemdan

**Affiliations:** 1 Department of Otorhinolaryngology, Faculty of Medicine, Mansoura University, El-Gomhoria Street, Mansoura, Egypt; 2 Department of Diagnostic Radiology, Faculty of Medicine, Mansoura University, El-Gomhoria Street, Mansoura, Egypt; 3 Department of Endocrinology, Diabetes and Metabolism, Faculty of Medicine, Mansoura University, El-Gomhoria Street, Mansoura, Egypt

**Keywords:** Endoscopic, Transcanal, Coblation, Glomus tympanicum

## Abstract

**Objective:**

To evaluate the feasibility of coblation in excision of glomus tympanicum tumors.

**Patients and methods:**

A retrospective study carried out over 28 patients with types I and II glomus tympanicum tumors according to GLASSCOCK-JACKSON classification. Preoperative radiological and endocrinal evaluation were performed. All patients underwent endoscopic transcanal excision of their glomus tympanicum tumors using coblation.

**Results:**

None of the patients developed recurrence during the 1-year follow up period proved radiologically. None of the patients developed facial palsy postoperatively. Differences between preoperative and postoperative dizziness and taste disturbance were statistically non-significant. Tinnitus disappeared completely in 22 patients postoperatively. A statistically significant reduction in Tinnitus Handicap Inventory (THI) after surgery was found. Statistically significant reductions in postoperative air conduction (AC) threshold and air bone gap (ABG) were recorded while bone conduction (BC) threshold showed statistically non-significant change.

**Conclusion:**

Coblation is an effective and safe tool in excision of glomus tympanicum tumors. Further studies comparing coblation with laser and piezosurgery are strongly recommended.

## Introduction

Glomus tympanicum is a benign vascular tumor arising from the adventitia of the tympanic plexus at the level of the promontory at the medial wall of the middle ear [1]. The commonest presentation is pulsatile tinnitus followed by hearing loss which may be conductive or mixed type [2]. Since it is a highly vascular tumor, the most troubling issue during surgical excision is the control of bleeding. Accordingly, different tools had been utilized to enhance hemostasis including, electrocautery, laser and piezosurgery.

Coblation is a modern technique that had been used in different ear, nose, and throat (ENT) surgeries including tonsillectomy, adenoidectomy, turbinate reduction, sleep surgeries and laryngeal microsurgeries. It is considered a type of electrosurgery acting through production of plasma field using radio-frequency energy and normal saline. This will result in low temperature and tissue disintegration [3].

The utilization of the coblation in ear surgery, including excision of glomus tympanicum, had not been discussed in the literature. This study, to our knowledge, is the first in the literatures that evaluated the feasibility of coblation in excision of glomus tympanicum. Moreover, it is the first study that reported the utilization of coblation in otologic surgery.

## Patients and methods

A retrospective study carried out on all patients with types I and II glomus tympanicum tumors who underwent endoscopic transcanal coblation excision of their glomus tumors in the period between November 2015 and November 2023 at our tertiary referral center. Patients’ medical records were collected and evaluated. Prior to study conduction, Institutional Ethics Committee approval was obtained (code: R.23.11.2393).

All patients were classified as type I or type II glomus tympanicum tumors according to GLASSCOCK-JACKSON classification. Type I described glomus tumor appearing as a small mass limited to promontory. Type II indicated that the tumor was completely filling a middle ear space. Patients with type III or type IV glomus tympanicum were excluded. Patients who missed to follow up with resultant incomplete medical records were also excluded.

**Fig. 1:**
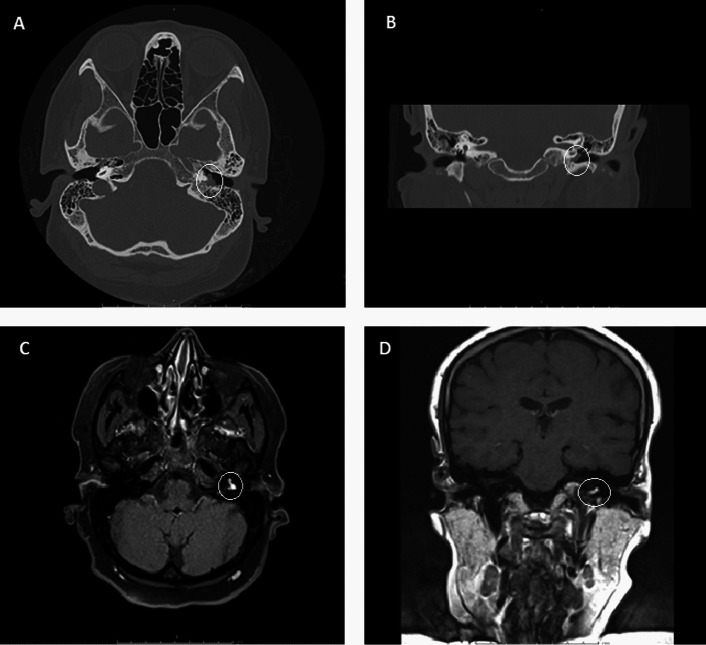
radiological evaluation of glomus tympanicum showing a left sided enhancing well-defined soft tissue mass (encircled) centered on the cochlear promontory. **A** HRCT axial view, **B** HRCT coronal view, **C** Gadolinium enhanced MRI axial view, **D** Gadolinium enhanced MRI coronal view

Radiological evaluation was performed using high resolution computed tomography scan (HRCT) and gadolinium-enhanced MRI with interpretation by our glomus team radiologist (second author). The aim of radiological evaluation was to confirm the diagnosis of glomus tumor by the presence of an enhancing soft tissue mass inside the middle ear. In addition, radiological evaluation enabled us to differentiate glomus tympanicum from both glomus jugulare and glomus jugulotympanicum. Moreover, detection of the tumor type; according to GLASSCOCK-JACKSON classification; could not be carried out without full radiological evaluation for tumor delineation and extension to surrounding structures (Fig. 1).

All involved cases then underwent a full endocrinal assessment by our glomus team endocrinologist (third author) aiming at detection of catecholamine secreting glomus tympanicum. A full history was taken with special focusing on hypertension, cardiac diseases, palpitation, diaphoresis, and diarrhea. The patients were then examined looking for wheezing, flushing, murmurs, and hypertension. Since biochemical assessment of catecholamine is affected by different kinds of drugs, all patients were asked about history of drug intake and if there was history of intake of any of these drugs, it was stopped for 2 weeks before biochemical analysis. Such drugs included: tricyclic antidepressants, phenoxybenzamine, labetalol, Monoamine oxidase inhibitors, sympathomimetics, caffeine, levodopa, carbidopa, cocaine, acetaminophen and buspirone. Biochemical analysis was done by measuring the level of plasma-free metanephrine while the patient was in supine position according to the endocrinal society guidelines which recommended measurement of either plasma-free metanephrine in supine position or urinary fractionated metanephrine [4]. If the level of plasma-free metanephrine was normal, surgery could be performed safely. If the level was elevated but < 4 times the normal level, surgery could also be performed but with intraoperative close monitoring of blood pressure. If the level was elevated ≥ 4 times the normal level, the patient was instructed to receive a combination of α and β blockers prior to surgery with intraoperative close monitoring of blood pressure.

In our institute, we use the exclusive endoscopic transcanal approach in management of all patients with types I and II glomus tympanicum tumors according to GLASSCOCK-JACKSON classification. Before November 2015, we utilized bipolar electrocautery in management of all types of glomus tympanicum tumors but since that date, we replaced the bipolar electrocautery with the coblation in all types of glomus tympanicum tumors.

**Fig. 2:**
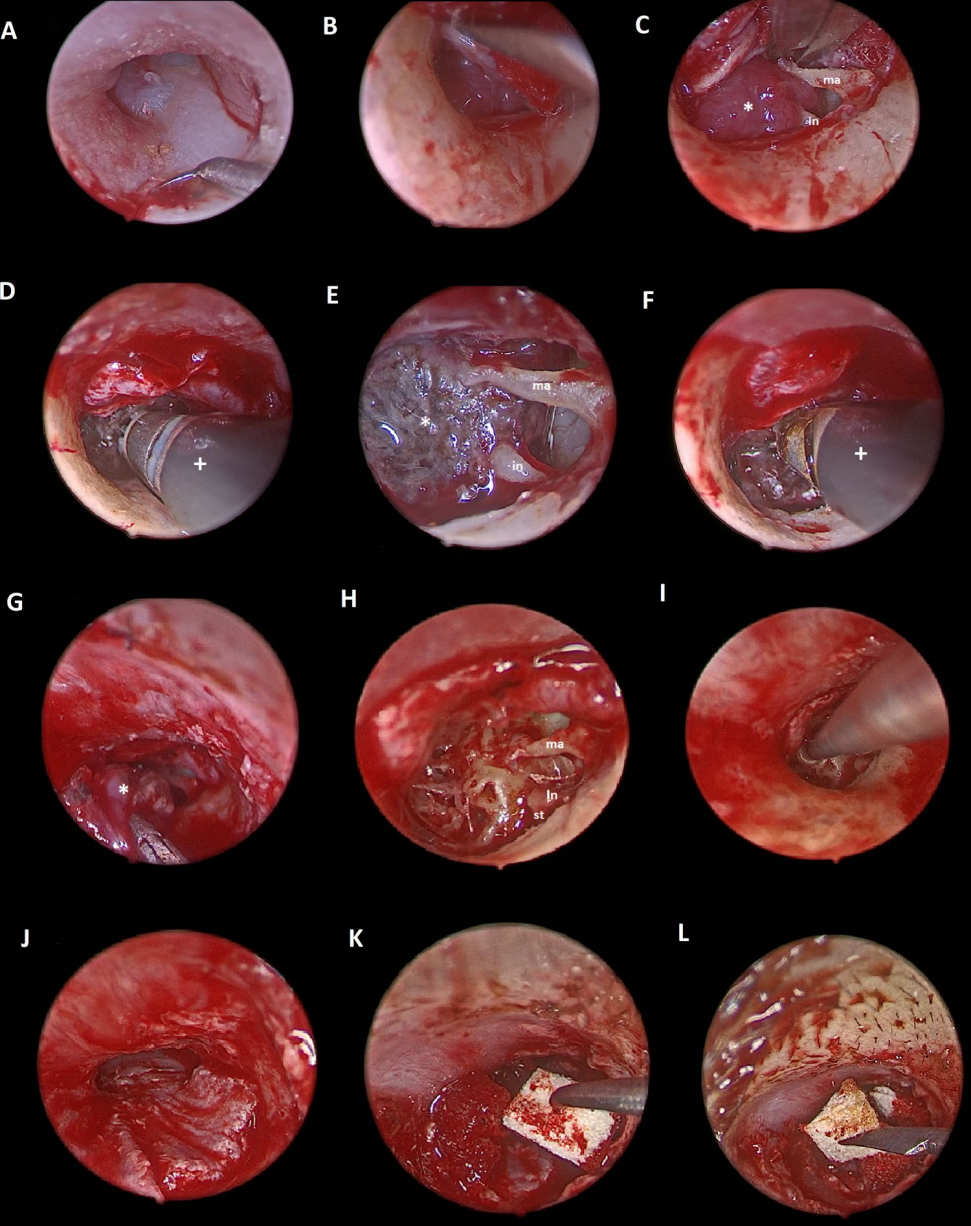
Surgical steps of endoscopic transcanal coblation excision of glomus tympanicum. **A** Skin incision of the external ear canal, **B** elevation of the annulus and entrance of the middle ear cavity, **C** separation of the tympanic membrane from the handle of malleus carefully (ma: malleus, in: incus, *: tumor), **D** coagulation of the tumor mass at the beginning (**+ **: coblation probe), **E** shrinkage of the tumor after coagulation (ma: malleus, in: incus, *: tumor), **F** dissection of the tumor from the surrounding bony walls with careful isolation of its feeding vessels using ablation hand in hand with coagulation (**+ **: coblation probe), **G** the tumor was fully excised (*: tumor), **H** clear tympanic cavity after tumor excision (ma: malleus, in: incus, st: stapes), **I** drilling of the site of origin of the tumor using diamond burr at low speed, **J** the tympanomeatal flap was repositioned, **K** small pieces of gelfoam were inserted to support the tympanomeatal flap, **L** filling the ear canal with gelfoam

All surgeries were performed under general anesthesia using 0° and 30° endoscopes, 4 mm diameter and 18 cm length (Karl Storz, Germany). Such endoscopes were connected to HD camera head attached to a monitor (Karl Storz, Germany). Four-quadrant injection of the skin of the external ear canal with a mixture of 2% lidocaine and 1/100,000 epinephrine was carried out at the beginning. Skin incision of the external ear canal was performed (Fig. 2A) followed by tympanomeatal flap elevation till the tympanic annulus which was elevated to enter the middle ear cavity (Fig. 2B). Careful dissection of the tympanic membrane was necessary with complete separation from the handle of malleus (Fig. 2C). Once the middle ear cavity was sufficiently exposed, dealing with the tumor mass was started. A Coblation probe (Procise™ MLW Wand) connected to Coblator II apparatus was utilized. Surgery started with coagulation of the tumor mass by coblation (Fig. 2D) to enhance tumor shrinkage (Fig. 2E). Dissection of the tumor from the surrounding bony walls with careful isolation of its feeding vessels was carried out using coblation (Fig. 2F). Small cottonoids soaked with 1/100,000 epinephrine were utilized to enhance hemostasis. The feeding vessels were then coagulated, and the tumor was fully excised (Fig. 2G) leaving clear tympanic cavity (Fig. 2H). Drilling of the site of origin of the tumor was done using diamond burr at low speed (Fig. 2I). The tympanomeatal flap was repositioned (Fig. 2J) and small pieces of gelfoam were inserted for support (Fig. 2K). The ear canal was finally filled with gelfoam (Fig. 2L).

Hearing was assessed according to American Academy of Otolaryngology head and neck surgery (AAOHNS) guidelines which recommended measurement of air conduction (AC) threshold, bone conduction (BC) threshold and air bone gap (ABG) for the frequencies 0.5,1, 2 and 3 kHz [5]. In all patients, bone conduction assessment was done with masking by employing narrow-band noise to the non-test ear to avoid cross-hearing. Tinnitus was assessed by Tinnitus Handicap Inventory (THI) which is a questionnaire that consists of 25 items about difficulties associated with tinnitus. In THI, the higher the score the higher the handicap. A follow up combined HRCT and gadolinium-enhanced MRI was done 1-year after surgery in all patients.

### Data collection and statistical analysis

Both preoperative and postoperative data were collected, organized, and analyzed. The collected data were displayed in the form of mean ± standard deviation (SD). Analysis was carried out using SPSS for Windows version 28 (Statistical Package for Social Sciences = SPSS Inc., Chicago, IL, USA). The paired t test was utilized to compare pre-operative with post-operative results. When *P* value was < 0.05, a statistically significant result was then considered.

## Results

Twenty-nine patients (of types I and II glomus tympanicum) underwent endoscopic transcanal coblation excision of their glomus tumors in the period between November 2015 and November 2023. One patient out of these 29 patients was missed to follow up 1 month after surgery and was excluded from the study. Accordingly, 28 (26 females and 2 males) were enrolled in the study. The mean age ± SD was 45.43 ± 8.62 years. The minimum and maximum ages were 32 and 65 years, respectively.

**Fig. 3:**
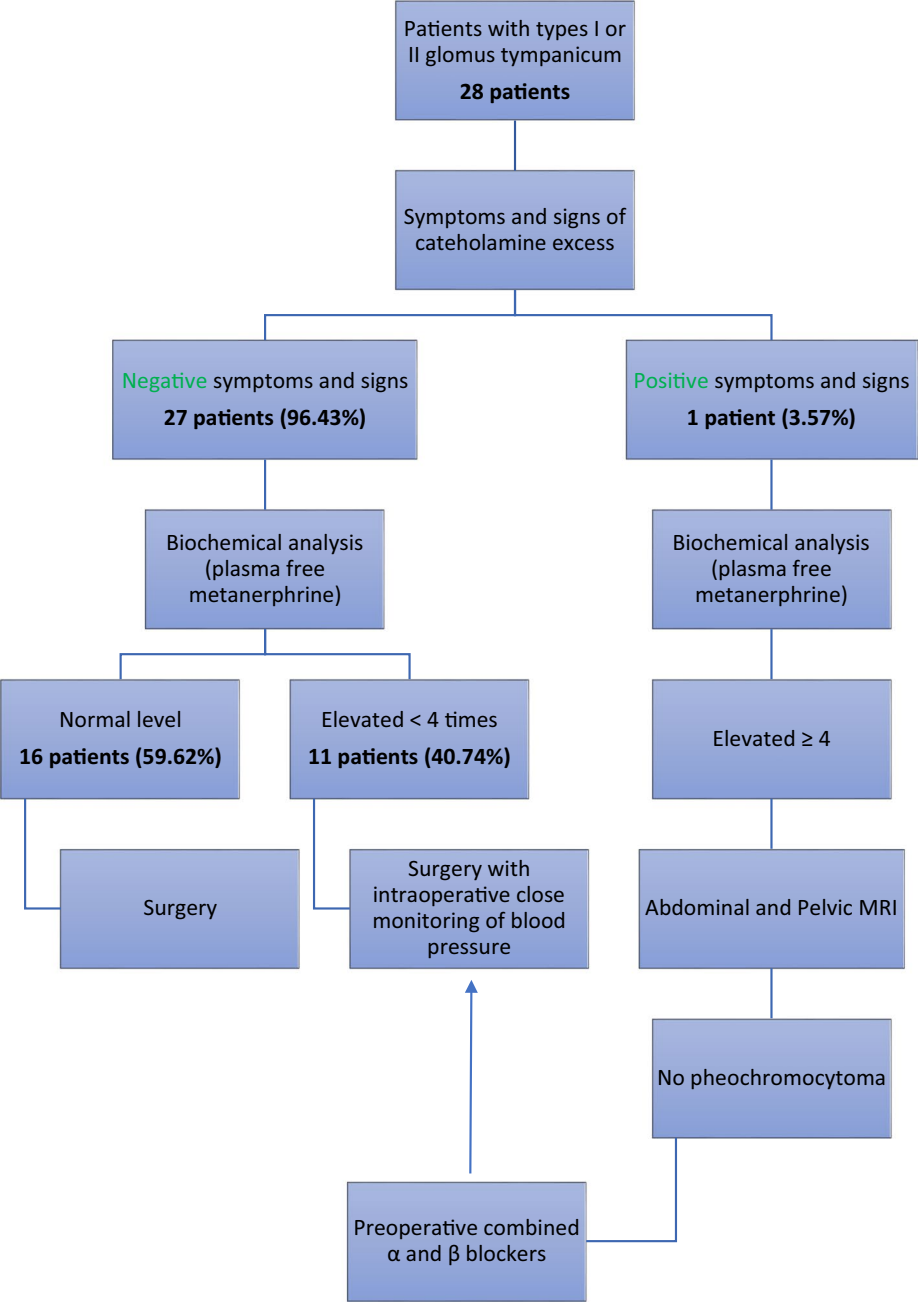
Study diagram of the retrospective cohort

As shown in Fig. 3, results of preoperative endocrinal evaluation showed that 27 out of the 28 included patients (96.43%) had no symptoms or signs of catecholamine excess. In these 27 patients, plasma-free metanephrine levels were normal in 16 patients (59.62%) and elevated in 11 patients (40.74%). However, such elevation was less than 4 times the normal level. Only 1 out of the 28 involved patients (3.57%) had symptoms and signs of catecholamine excess. Plasma-free metanephrine level of this single patient was elevated more than 4 times the normal level. This patient underwent abdominal and pelvic MRI but there was no pheochromocytoma. The recommendation by our endocrinologist was to administer combined alpha and beta blockers preoperatively which were administered for 2 weeks prior to surgery.

All patients underwent complete endoscopic transcanal excision of their glomus tympanicum tumors using coblation. None of the patients developed recurrence during the 1-year follow up period proved radiologically with combined HRCT and gadolinium-enhanced MRI. None of the patients had preoperative facial palsy or developed facial palsy postoperatively. Regarding taste, none of the patients had preoperative taste disturbance. After surgeries, 3 patients (10.71%) complained from postoperative taste disturbance. However, such postoperative change in taste was statistically non-significant (*P* value 0.083). Only 2 patients suffered from dizziness preoperatively which increased to 5 patients postoperatively with statistically non-significant difference (*P* value 0.424).

Preoperatively, all 28 patients had tinnitus which disappeared completely in 22 patients postoperatively. The remaining 6 patients described an overall reduction in their tinnitus severity although it was still present. The mean preoperative THI ± SD was 43.86 ± 14.02. Postoperatively, a reduction in the mean THI ± SD into 3.07 ± 6.07 was found. Such reduction in THI after surgery was statistically significant (*P* value < 0.001) (Table 1).

**Table 1:** Differences between preoperative and postoperative THI and hearing results

	Preoperative	Postoperative	*P* value
Tinnitus Handicap Index (THI)	43.86 ± 14.02	3.07 ± 6.07	< 0.001
Hearing			
AC	40.02 ± 11.50	31.50 ± 8.53	0.001
BC	16.92 ± 2.94	16.43 ± 3.43	0.36
ABG	23.12 ± 10.62	15.07 ± 6.27	0.001
SRT	39.11 ± 11.47	30.89 ± 8.82	0.002
SDS	96.07 ± 6.41	96.43 ± 6.33	0.33

The mean preoperative AC threshold ± SD was 40.02 ± 11.50 dB with postoperative reduction into 31.50 ± 8.53 dB. Such reduction was statistically significant (*P* value 0.001). The mean pre and postoperative BC thresholds ± SD were 16.92 ± 2.94 and 16.43 ± 3.43, respectively with statistically non-significant difference (*P* value 0.36). The mean preoperative ABG ± SD was 23.12 ± 10.62 dB. Reduction in the postoperative ABG into 15.07 ± 6.27 dB was found which was statistically significant (*P* value 0.001). The mean pre- and postoperative SRTs were 39.11 ± 11.47 and 30.89 ± 8.82, respectively with statistically significant difference (*P* value 0.002). The mean pre- and postoperative SDSs were 96.07 ± 6.41 and 96.43 ± 6.33, respectively with statistically non-significant difference (*P* value 0.33) (Table 1).

## Discussion

In glomus tumors, preoperative combined HRCT and MRI are mandatory for confirming diagnosis, delineation of the tumor extensions and tumor staging. Classically, HRCT allows bony delineation of the extent of glomus tumors within the temporal bone. Gadolinium enhanced MRI allows for detection of intracranial and infratemporal extension of the tumors [6].

Screening for catecholamines is essential in all cases of glomus tumors. This could be explained by the fact that almost all paragangliomas have the potential to secrete catecholamines although the manifestations are apparent in only 1–3% of cases. For manifestations to appear, a four to fivefold increase in the catecholamines levels should occur. Accordingly, it is unsurprising to find that several cases with glomus tumors will have low levels of catecholamines despite being asymptomatic [7–10]. In this study, only 1 patient out of the 28 involved patient (3.57%) had symptoms and signs of catecholamines excess. This was comparable to the previously published studies which reported that symptoms producing glomus tumors are rare [7–10]. Although 96.43% of the involved cases were free from symptoms and signs of catecholamines excess, plasma-free metanephrine level was elevated < 4 times the normal level in 40.74% of these patients. This agreed with previously published studies which necessitated the elevation of catecholamines for ≥ 4 times to produce symptoms [7–11].

Surgical excision is the standard treatment of glomus tympanicum. The challenge during glomus tympanicum surgery is to obtain adequate exposure and good hemostasis. Accordingly, different facilities had been utilized to achieve good hemostasis including bipolar electrocautery, laser and piezosurgery.

Bipolar electrocautery is the traditional tool for glomus tympanicum excision. It was described in different studies [12–14]. Although it allows adequate hemostasis, the generated heat may burn the skin of the external ear canal or affect the facial nerve. In addition, transmission of such generated heat into the inner ear was inevitable.

The utilization of laser in glomus surgery; including Diode, KTP and Nd:YAG lasers; had been described in different studies. The laser allows an excellent coagulation of the highly vascular glomus tumors with minimal trauma to the healthy tissues like ossicles and facial nerve. Moreover, the strong coagulative power offered by laser allows shrinkage of the tumor with resultant enhanced exposure [6]. The main disadvantage of laser is the liability for development of sensory neural hearing loss (SNHL) owing to energy transmission via the round window with resultant damage to the hair cells [11].

Different case reports about laser excision of glomus tumors were published. Robinson et al. used Nd:YAG laser in a 60-year-old female with large glomus tympanicum tumor and obtained good hemostasis and complete tumor removal [11]. Molony et al. used KTP laser in a 39-years old female with glomus tumor and achieved complete tumor excision with minimal blood loss [15]. Another 2 case reports by Chen et al. and Alkhedr et al. utilized Diode laser in 37- and 56-year old females respectively and reported good hemostasis and complete tumor removal [16, 17]. Durvasula et al. assessed the long-term results of laser treatment of glomus tympanicum among 9 cases (6 cases were managed using Diode laser and 3 cases using KTP laser). They found no tumor recurrence and no complications in the postoperative period [6]. Another case series was performed by Noel and Sajjadi who utilized KTP laser in management of 5 cases of glomus tympanicum and obtained successful tumor removal in all patients with ideal exposure and hemostasis [18].

Piezosurgery is a new technique that cut the bone selectively without damage to the non-mineralized tissues. It utilizes the microvibrations of scalpels at ultrasonic frequencies. It offers a bloodless field with excellent visibility and preservation of soft tissues [19]. Salami et al. published a case series of 10 patients with glomus tympanicum who underwent successful excision of their tumors using piezosurgery with absent side effects together with protection of the anatomical structures [20]. Another study by O’Connell et al. was conducted among three cases (2 cases with glomus tympanicum and 1 case with small glomus jugulare) and reported complete tumor excision without complications [21].

Coblation is a modern surgical tool that joins the procedures of dissection, coagulation, hemostasis, irrigation and suction in a single device. In addition, the electrode is utterly flexible enhancing its operability [22].

Coblation is based on utilization of bipolar radiofrequency in creation of a current through saline solution leading to ionization of the particles of saline. Such ionization is then transmitted into the tissues molecular bonds leading to ablation. The main advantage of the coblation over electrocautery is the lesser temperature required to produce ablation which is between 40 and 70 °C in coblation while it can reach up to 400 °C in electrocautery. Such lower temperature reduces tissue damage and postoperative pain [23, 24].

Coblation has been utilized in various ENT surgical procedures including, adenotonsillectomy, inferior turbinate reduction and various microlaryngeal surgeries. However, its utilization in ear surgery had not been reported in the literatures. Due to its advantages of lower temperature generated and combining the procedures of dissection, coagulation, hemostasis, irrigation and suction in a single device, we proposed that its utilization in ear surgery, especially the highly vascular glomus tympanicum, will be effective.

In this study, the mean age of the included cases was 45.43 ± 8.62 years. There was female predominance over male. These finding agreed with the previously published studies [25, 26].

No tumor recurrence was encountered within the 1-year follow-up period proved radiologically with follow up HRCT and gadolinium-enhanced MRI. Tinnitus was the most common symptom encountered in all involved cases. Tinnitus was assessed using THI. In this study, a statistically significant reduction in the postoperative THI in comparison to the preoperative results was encountered. Such finding ensured the efficacy of coblation technique in management of glomus tympanicum. Regarding dizziness, no statistically significant difference between pre- and postoperative dizziness indicating that coblation technique had mostly no harmful effect on the inner ear. Such finding may be explained by the lesser temperature generated by the Coblator. Moreover, no significant change in the taste sensation postoperatively in comparison to the preoperative results which indicated the safety of this technique over the chorda tympani. Hearing evaluation was another important point of interest. A significant reduction in the postoperative AC and ABG was found ensuring the efficacy of this technique in improving hearing. Moreover, no significant change in postoperative BC in relation to the preoperative results. Again, this could emphasize the safety of coblation technique over the inner ear which could be explained by the lesser temperature generated which is not transmitted to the cochlea. Such result might give an advantage for coblation technique over laser and bipolar electrocautery. No disruption of the integrity of the ossicles or facial nerve during surgery. This could be explained by the tumor contracture due to the effect of coblation which enhanced exposure of the tumor surroundings and reduced manipulation of the important structures including facial nerve and ossicles.

There is no cutoff time for recurrence of glomus tumor. Sanna et al. [27] found that majority of recurrences occurs after at least 5 years. Another study by Contrera et al. [28] estimated that the median time for recurrence was 18.4 years following treatment. Thus, the recommendation of the Endocrinal Society was to follow up the patients for at least 10 years after treatment [29].

The main limitations of this study were the short follow up period and the small number of the study population. We recommend further studies with long follow-up period and larger study population. In addition, we encourage further studies comparing coblation technique with laser and piezosurgery in management of glomus tympanicum.

## Conclusion

Coblation is an effective and safe tool in excision of glomus tympanicum tumors. Further studies comparing coblation with laser and piezosurgery are strongly recommended. In addition, we recommend further studies with long follow up period and larger study population.

## Data Availability

The datasets generated during and/or analysed during the current study are available from the corresponding author on reasonable request.
